# Supplementary material for: β-Lactoglobulin-Modified Mesoporous Silica Nanoparticles: A Promising Carrier for the Targeted Delivery of Fenbendazole into Prostate Cancer Cells
Source: Pharmaceutics. 2022 Apr 18;14(4):884. doi: 10.3390/pharmaceutics14040884 (PMC9024783; doi:10.3390/pharmaceutics14040884)
Supplement: Supplementary file 1 [file pharmaceutics-14-00884-s001.zip › pharmaceutics-1681569-supplementary.pdf]

pharmaceutics

MDPI

Supplementary Materials: β-Lactoglobulin-Modified Mesoporous Silica Nanoparticles: A Promising Carrier for the Targeted Delivery of Fenbendazole into Prostate Cancer Cells

Maedeh Koohi Moftakhari Esfahani, Seyed Ebrahim Alavi, Peter J. Cabot, Nazrul Islam and Emad L. Izake

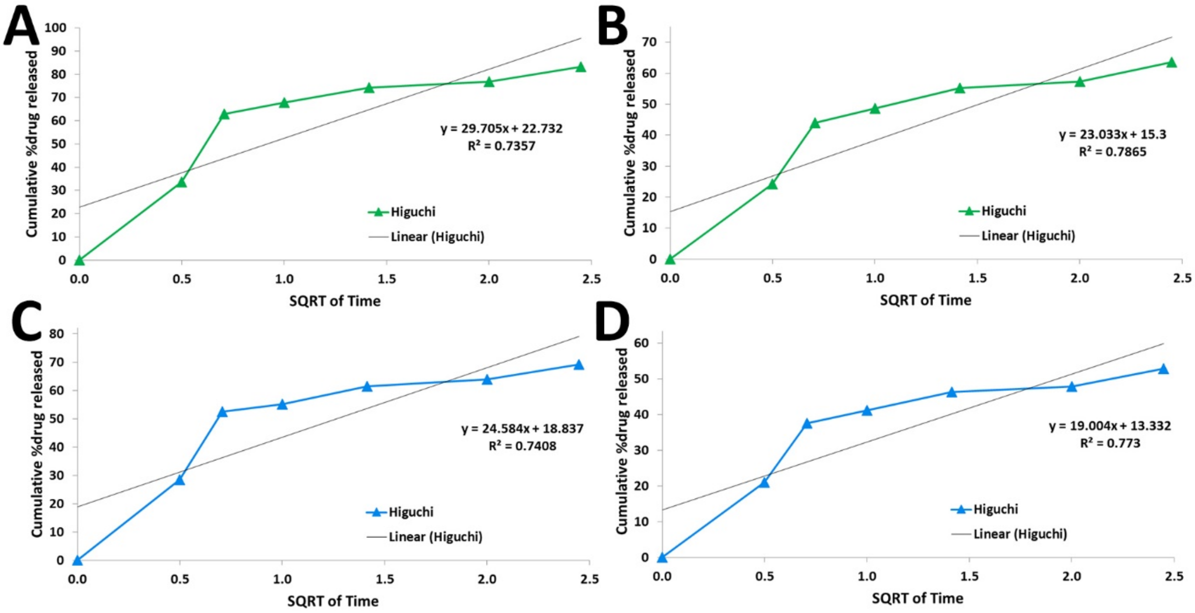

Figure S1. The Higuchi model of FBZ release from (**A**) FBZ-MCM (pH1.2), (**B**) FBZ-MCM (pH6.8), (**C**) FBZ-MCM-BLG (pH1.2), and (**D**) FBZ-MCM-BLG (pH6.8). Results are expressed as mean ± SD of three independent experiments.

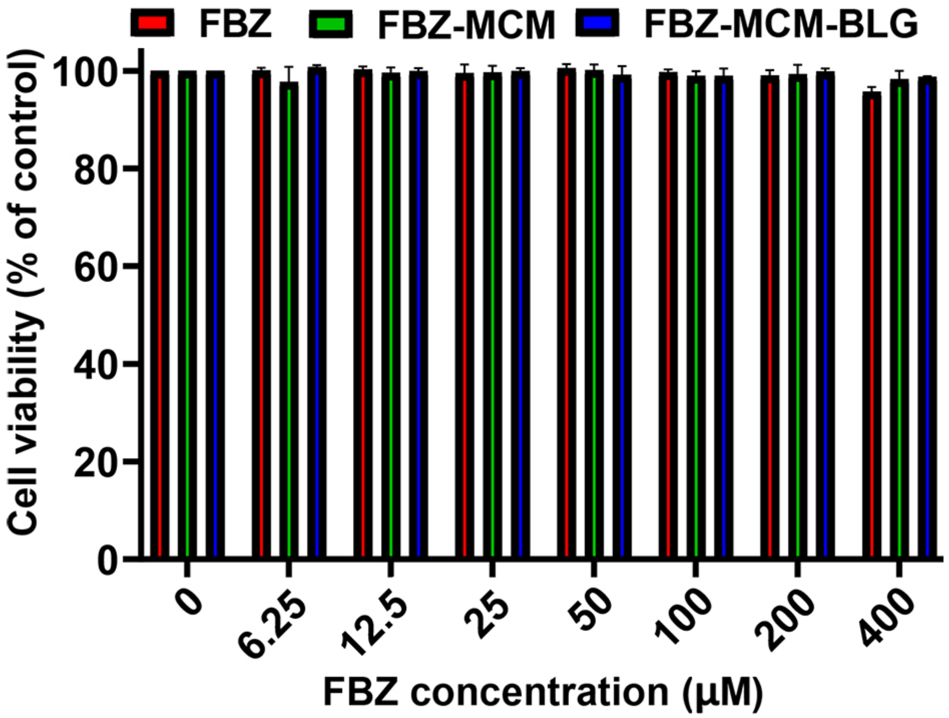

Figure S2. Viability effects of FBZ-MCM and FBZ-MCM-BLG nanoparticles, compared to FBZ, on the human embryonic kidney 293 (HEK-293) cells after 48 h incubation. The data are expressed as mean ± SD (n = 3).
